# Supplementary material for: Analysis of PPARγ Signaling Activity in Psoriasis
Source: Int J Mol Sci. 2021 Aug 10;22(16):8603. doi: 10.3390/ijms22168603 (PMC8395241; doi:10.3390/ijms22168603)
Supplement: Supplementary file 1 [file ijms-22-08603-s001.zip › Supplemental materials_Analysis of PPARg signaling activity in psoriasis/Pathway models/Models images and html files/PPARG inhibits phenotypes assotiated with psoriasis/244988.html]

cardiac remodeling


# Disease cardiac remodeling

|  |  |
| --- | --- |
| URN | urn:agi-disease:cardiac%20remodeling |
| Total Entities | 2 |
| Connectivity | 2996 |
| Name | cardiac remodeling |

---

|  |  |
| --- | --- |
| ChildConcepts | atrial remodeling |
|  | ventricular remodeling |

---

|  |  |
| --- | --- |
| Pathway | Myocardial Infarction |
|  | PPARG inhibits phenotypes assotiated with psoriasis |
|  | Neighbors of cardiac remodeling |

---

|  |  |
| --- | --- |
| MedScan ID | 9017515 |

---

|  |  |
| --- | --- |
| Alias | cardio-vascular remodeling |
|  | myocardial remodeling |
|  | cardiovascular remodeling |
|  | myocardium remodeling |
|  | remodeling of the myocardium |
|  | changes of the cardiovascular |
|  | remodeling of the ventricle |
|  | heart remodeling |
|  | cardiac remodeling |
|  | cardiac valve remodeling |
|  | cardio-vascular changes |
|  | remodeling in the heart |
|  | cardiovascular changes |

---
